# Distributed Compressive Sensing for Wireless Signal Transmission in Structural Health Monitoring: An Adaptive Hierarchical Bayesian Model-Based Approach

**DOI:** 10.3390/s23125661

**Published:** 2023-06-17

**Authors:** Zhiwen Wang, Shouwang Sun, Yiwei Li, Zixiang Yue, Youliang Ding

**Affiliations:** 1 Key Laboratory of Concrete and Pre-Stressed Concrete Structures of the Ministry of Education, Southeast University, Nanjing 210096, China; 2 YunJi Intelligent Engineering Co., Ltd., Shenzhen 518000, China

**Keywords:** structural health monitoring, distributed compressive sensing, hierarchical Bayesian model, wireless signal transmission, Laplace prior

## Abstract

Signal transmission plays an important role in the daily operation of structural health monitoring (SHM) systems. In wireless sensor networks, transmission loss often occurs and threatens reliable data delivery. The massive amount of data monitoring also leads to a high signal transmission and storage cost throughout the system’s service life. Compressive Sensing (CS) provides a novel perspective on alleviating these problems. Based on the sparsity of vibration signals in the frequency domain, CS can reconstruct a nearly complete signal from just a few measurements. This can improve the robustness of data loss while facilitating data compression to reduce transmission demands. Extended from CS methods, distributed compressive sensing (DCS) can exploit the correlation across multiple measurement vectors (MMV) to jointly recover the multi-channel signals with similar sparse patterns, which can effectively enhance the reconstruction quality. In this paper, a comprehensive DCS framework for wireless signal transmission in SHM is constructed, incorporating the process of data compression and transmission loss together. Unlike the basic DCS formulation, the proposed framework not only activates the inter-correlation among channels but also provides flexibility and independence to single-channel transmission. To promote signal sparsity, a hierarchical Bayesian model using Laplace priors is built and further improved as the fast iterative DCS-Laplace algorithm for large-scale reconstruction tasks. Vibration signals (e.g., dynamic displacement and accelerations) acquired from real-life SHM systems are used to simulate the whole process of wireless transmission and test the algorithm’s performance. The results demonstrate that (1) DCS-Laplace is an adaptative algorithm that can actively adapt to signals with various sparsity by adjusting the penalty term to achieve optimal performance; (2) compared with CS methods, DCS methods can effectively improve the reconstruction quality of multi-channel signals; (3) the Laplace method has advantages over the OMP method in terms of reconstruction performance and applicability, which is a better choice in SHM wireless signal transmission.

## 1. Introduction

Structural health monitoring (SHM) has become a rapidly developing technology in the last decade, aimed at the damage detection and condition assessment of structures with sensing techniques and structural characteristics analysis [1,2,3,4,5]. Data transmission is an important step in SHM applications, which is based on the hardware implementation of sensor networks. Compared with traditional wired sensing, wireless sensing has unique advantages, leading to the attractive prospect of monitoring large civil infrastructures [3,6,7,8]. However, packet loss is a common problem in wireless signal transmission due to a range of reasons, including radio interference, large transmission distances, and hardware failure [9,10,11]. In addition, to comprehensively monitor the dynamic response of a structure under external excitations, a great number of sensors are deployed for the excessive collection of data. For example, sensors are usually sampled at higher frequencies than the actual signal frequency. The huge amount of data will cost large storage space and energy consumption during transmission, increasing the workload of the sensor networks [1,3,12]. To tackle these problems, traditional communication technologies have been applied in SHM. Data compression attempts to exclude redundant data before transmission, thus improving power efficiency [13,14,15]. On the other hand, several reliable communication protocols have been widely adopted to cope with data loss by retransmitting the lost packets [16,17,18,19]. However, conventional compression approaches are extravagant as the over-captured data still requires plenty of memory while the redundant data are selectively discarded afterward. Meanwhile, the retransmission-based methods greatly limit communication efficiency and cannot fundamentally mitigate the impact of missing data.

Compressive Sensing (CS) provides a new perspective for wireless signal transmission. In the CS-based technique, instead of transmitting the original signal, the measurements obtained by projecting the original signal into a low-dimensional and incoherent space are recorded and transmitted [11,12]. The length of the measured vector is generally reduced, which is equivalent to data compression or partial data loss during transmission. Utilizing the sparsity of the signal on some basis, the original signal can be effectively reconstructed from the received incomplete measurements [20,21]. This indicates that CS can not only maintain robustness to data loss but also facilitate data compression for energy efficiency. In SHM, related work has been carried out on vibration signals with high sparsity in the frequency domain [22,23,24,25,26,27,28,29]. Bao et al. [22], O’Connor et al. [23], Klis et al. [24], Jayawardhana et al. [12], and Wan et al. [25] implemented CS-based data compression and reconstruction on different types of vibration signals. Bao et al. [9,10,11] and Li et al. [26] proposed data loss recovery approaches according to the CS theory. Huang et al. [27,28] innovatively applied Bayesian compressive sensing (BCS) to SHM. Recently, an adaptive CS method incorporating deep learning has been explored for vibration data transmission in high-speed railroads with ideal results [29].

On the other hand, improving the signal quality during transmission can further enhance the data compressibility and resistance to packet loss. A common phenomenon in SHM is that there is a degree of spatial or temporal correlation among structural responses acquired from the sensor network, especially for sensors distributed on an identical structure with similar loading conditions. Some studies have achieved high data availability by exploiting the correlation among multiple sensors. For example, Zhang et al. [30] restored missing stress data with an interpolation method based on the correlation of multi-sensor stress changes; Chen et al. [31,32,33,34] developed a distribution regression approach for missing data imputation; and Zhang et al. [35] presented a Bayesian dynamic regression method to capture the relationship among sensors and reconstruct the missing data. However, the multi-sensor correlation modeling in the above studies depends on the long-term and cumulative monitoring of data. In wireless transmission, signals should be sent and received in real time, and reconstruction tasks are usually conducted on segment-wise measurements with a fixed length. The different application scenarios lead to the fact that the above methods are not suitable for enhancing the reconstruction quality in signal transmission.

Fortunately, a novel technique called Distributed Compression Sensing (DCS) has been gradually developed in the field of CS [36,37,38,39,40,41,42,43,44,45]. Compared with CS for the basic single measurement vector (SMV), DCS can take advantage of the inter-correlation of multiple measurement vectors (MMV) to jointly recover the multi-channel signals with approximate sparse patterns, thus improving the reconstruction performance [36,37,38,39]. The DCS methods are usually extended from the original CS methods and can be broadly classified as greedy methods, such as Simultaneous Orthogonal Matching Pursuit (SOMP) [40], iterative reweighted methods [37,41], and Bayesian approaches, such as Bayesian compressive sensing [42], Sparse Bayesian Learning (SBL) [43,44], and the Laplace method [45] under MMV cases. In SHM, some scholars have preliminarily verified the effectiveness of DCS [46,47,48,49,50,51,52]. Bao et al. [46,47] proposed a group sparse optimization algorithm on the basis of the group sparsity of structural vibration data; Huang et al. [48,49] and Wan et al. [50] applied multi-task Bayesian methods to data loss recovery and structural damage diagnosis; and Amini et al. [51] used the algorithm of DCS-SOMP to recover multi-channel signals with different data loss patterns. Recently, Wan et al. [52] developed an improved complex multi-task Bayesian compressive sensing approach that allows the joint reconstruction of vibration signals on the discrete Fourier basis. The promising results show the great potential of DCS for wireless signal transmission in SHM. However, it should be noted that (1) the issues of data compression and data missing recovery are mostly considered separately in the above studies, which lack discussion of their transforming relationship and comprehensive influence on wireless transmission and (2) the aforementioned research mostly concentrates on the underlying commonality across sensors while ignoring the transmission independence of each channel. In practical engineering, the compression, loss, and reconstruction scenarios of the signal from each channel are probably not identical, which causes difficulties in the application of DCS.

Given the limitations of previous studies, this paper explores the application of DCS techniques to SHM wireless transmission. The outcomes can be summarized as follows:

(1) A comprehensive DCS framework for wireless signal transmission is constructed, incorporating the process of data compression and transmission loss together. Unlike the basic DCS formulation, the proposed framework starts from practical necessity, which can not only activate the connection among the channels but also provide flexibility and independence to single-channel transmission. Specifically, the scheme enables a joint reconstruction of MMV with the same or even different compression and loss rates by using a unique sensing matrix for each channel.
(2) Considering that common priors in the Bayesian framework can be set flexibly to facilitate multi-task information sharing, a hierarchical Bayesian model is applied for multi-channel signal reconstruction. To strengthen the sparsity constraint on SHM signals, Laplace priors are imposed on sparse vectors. In addition, an efficient iterative algorithm based on a modified sparse regression model, called Fast DCS-Laplace, is employed to improve the computation efficiency in the face of large-scale problems.
(3) Vibration signals collected in real-life SHM systems with spatial or temporal correlations are used to simulate the whole process of wireless transmission and test the algorithm’s performance. In addition, a comparison with the DCS-SOMP algorithm that has recently been applied in SHM is carried out under the proposed DCS framework to prove the superiority of Fast DCS-Laplace.

This paper mainly involves three parts. The DCS methodology is presented in Section 1. Section 2 is the case validation using the dynamic displacement data from Lieshihe Highway Bridge and accelerations from Dashengguan High-Speed Railway Bridge, respectively. Section 3 is the conclusion. In this study, only vibration signals in SHM are considered because they are sparse enough in the frequency domain to satisfy the prerequisites of sparse reconstruction.

## 2. Methodology

### 2.1. General CS-DCS Framework

Compressive sensing (CS) [20,21], also known as compressive sampling or sparse signal recovery, is an effective approach for finding sparse solutions to underdetermined linear systems. In the general CS framework, *M* random measurements are acquired from the original signal $u\in {\mathbb{R}}^{N\times 1}$ M≤N as

(1) v=Φu

where $v\in {\mathbb{R}}^{M\times 1}$ is the available measured vector and $\Phi \in {\mathbb{R}}^{M\times N}$ is the projection matrix. To uniquely recover the unknown u given v and Φ, u should be sparse on a given basis Ψ as

(2) u=Ψw

where w is the *N*-dimensional sparse vector. The basis Ψ can be an N×N orthogonal matrix or an $N\times N_{1}$ redundant matrix where $N<N_{1}$ [53]. Substituting (2) into (1) yields

(3) v=ΦΨw=Aw

where A is an M×N sensing matrix. The basic model is usually called the single measurement vector (SMV) model as there is only a single measured vector. In distributed compressive sensing (DCS) [36,37,38,39], multi-channel sparse vectors ${\left\{w_{i}\right\}}_{i=1,2,\ldots ,L}$ can be jointly recovered from the corresponding set of *L*-measured vectors ${\left\{v_{i}\right\}}_{i=1,2,\ldots ,L}$. The framework has been extended to the multiple measurement vector (MMV) model given by

(4) V=AW

where $V≜[v_{1},v_{2},\ldots ,v_{L}]$ and $W≜[w_{1},w_{2},\ldots ,w_{L}]$ are the measured matrix and sparse matrix, respectively, with each column representing a possible signal channel. It is assumed that W should be jointly sparse (i.e., indexes of nonzero entries in each channel are identical) [37,44]. By exploiting the correlation among channels, the reconstruction performance of MMV models can be greatly enhanced compared with the SMV cases [40,41,42,43,44,45].

### 2.2. DCS Framework in SHM Wireless Transmission

#### 2.2.1. Stage 1: Data Compression

In the DCS framework, data compression is achieved through the transformation of original signals into projected signals. According to (1), the original signal of each channel ${\left\{u_{i}\in {\mathbb{R}}^{N\times 1}\right\}}_{i=1,2,\ldots ,L}$ is projected separately into the incoherent space to obtain the measurement vector ${\left\{v_{i}\in {\mathbb{R}}^{M_{i}\times 1}\right\}}_{i=1,2,\ldots ,L}$ using the corresponding projection matrix ${\left\{\Phi_{i}\in {\mathbb{R}}^{M_{i}\times N}\right\}}_{i=1,2,\ldots ,L}$

(5) $$v_{i}=\Phi_{i}u_{i}$$

The commonly used projection matrices include Gaussian- or Bernoulli-distributed random matrices [21]. However, in the wireless sensor network of SHM, the storage space, computational power, and energy consumption of the sensor node are always limited, which can cause great difficulties in the projection matrix embedding. To address this issue, a unique sampling approach called random demodulator (RD) is adopted to provide sufficient efficacy and efficiency for SHM data acquisition [11,54]. In this method, the projection matrix can be determined by multiplying two matrices, the demodulation matrix $D\in {\mathbb{R}}^{N\times N}$ and the sampling matrix $S_{i}\in {\mathbb{R}}^{M_{i}\times N}$, respectively. By adjusting the length and position of sliding bands in the sampling matrix, the signal compression ratio and the data loss redundancy can be jointly controlled [11]. In addition, a permutation matrix $P_{i}\in {\mathbb{R}}^{M_{i}\times M_{i}}$ is additionally multiplied to further reduce the coherence and to ensure robustness to continuous data loss [51]. Finally, the projection matrix for multi-channel signal compression can be expressed as

(6) $$\Phi_{i}=P_{i}S_{i}D$$

#### 2.2.2. Stage 2: Data Loss in Transmission

After data compression, the projected signal $v_{i}$ will be divided into multiple packets and transmitted wirelessly one by one. During this process, each channel may suffer varying degrees of data loss and the base station will receive *L* incomplete measured vectors ${\tilde{v}}_{i}\in {\mathbb{R}}^{{\tilde{M}}_{i}\times 1}$. Therefore, (5) would be rewritten as

(7) $${\tilde{v}}_{i}={\tilde{\Phi }}_{i}u_{i}$$

where ${\tilde{\Phi }}_{i}\in {\mathbb{R}}^{{\tilde{M}}_{i}\times N}$ is a submatrix of the original projection matrix. Since the indexes of packets received in each channel are recorded, the ${\tilde{\Phi }}_{i}$ can be obtained by extracting the corresponding rows of $\Phi_{i}$.

#### 2.2.3. Stage 3: Data Reconstruction

The signal reconstruction of multiple channels can be carried out simultaneously once measurements are received at the base station. Since the vibration signal in SHM is generally sparse in the frequency domain, the Discrete Cosine Transform (DCT) basis is used as Ψ in this study [55]. Substituting (2) into (7) yields

(8) $${\tilde{v}}_{i}={\tilde{\Phi }}_{i}\Psi w_{i}={\tilde{A}}_{i}w_{i}$$

where ${\tilde{A}}_{i}$ is an ${\tilde{M}}_{i}\times N$ sensing matrix. It can be observed that there are significant differences between the MMV models in (8) and (4). In (8), the length of the measured vectors ${\left\{{\tilde{v}}_{i}\right\}}_{i=1,2,\ldots ,L}$ can be different, which means that the joint sparsity property of multiple channels is probably not strictly satisfied (i.e., the number and locations of nonzero entries from each channel are not completely the same); the sensing matrix ${\tilde{A}}_{i}$ in each channel can be different, which means reconstruction tasks for all channels are distinct and independent in many cases. Therefore, to overcome the non-uniform transmission scenarios across MMV, the DCS scheme should not only achieve the joint recovery of multi-channel signals more practically but also provide flexibility and independence to single-channel reconstruction.

Hierarchical Bayesian models are one of the most important DCS methods [42,45,49]. Among the MMV algorithms, Bayesian methods usually have high reconstruction performance and computation efficiency and can provide an estimation of the reconstruction uncertainty from a probabilistic perspective [42,43,44,45]. More importantly, the flexible setting of common priors can facilitate information sharing of multiple reconstruction tasks while maintaining their individuality, which is greatly beneficial for the implementation of the MMV model in (8). Given the distinct advantages, an improved version of the hierarchical Bayesian model named DCS-Laplace is constructed in this study. The algorithm will be discussed in the following subsections.

**Figure 1:**
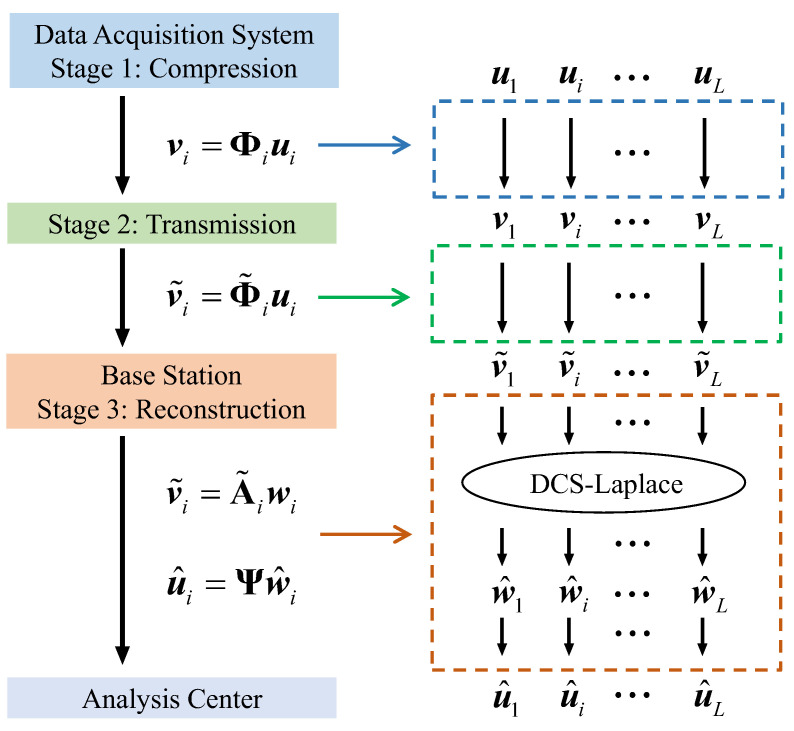
Workflow of the proposed DCS framework for wireless signal transmission in SHM.

After obtaining the estimated sparse vector ${\hat{w}}_{i}$, the recovered vibration signal ${\hat{u}}_{i}$ can be calculated by (2). The flow chart of the proposed DCS Framework for SHM wireless transmission is shown in Figure 1.

### 2.3. DCS-Laplace for Multi-Channel Signal Recovery

#### 2.3.1. Hierarchical Bayesian Modelling Using Laplace Priors

As a starting point for many CS sparse recovery algorithms, w in (3) is generally approximated by solving the following *l*_1_ regularized formulation [20,56]

(9) $$\hat{w}=\arg\underset{w}{\min}\left\{{\left\|\tilde{v}-\tilde{A}w\right\|}_{2}^{2}+\kappa {\left\|w\right\|}_{1}\right\}$$

where κ is the regularization coefficient that controls the sparsity of the formulation. From the Bayesian perspective, the mapping $\tilde{v}\to w$ can be converted into a sparse linear regression problem, where the unknown parameters can be considered as random quantities with prior knowledge represented by specified probability distributions [57,58]. The Gaussian likelihood function for the weights w under the observed vector $\tilde{v}$ is

(10) $$p\left(\tilde{v}|w,\beta \right)=N\left(\tilde{v}|\tilde{A}w,\beta^{-1}\right)$$

where $\beta =1/\sigma^{2}$ is the unknown precision in the regression task. The w is then assigned a prior distribution p(w|γ) to model its sparsity. It can be observed that the properties of the above distributions depend on the parameters γ and β, which are called hyperparameters, and the prior distributions are called hyperpriors. A Gamma prior is usually placed on β as

(11) $$p\left(\beta |a,b\right)=\Gamma \left(\beta |a,b\right)=\frac{b^{a}}{\Gamma \left(a\right)}\beta^{a-1}\exp\left(-b\beta \right)$$

In the framework of a relevance vector machine (RVM) or sparse Bayesian learning (SBL) [59], the prior distribution of w is expressed as the product of N zero-mean Gaussian distributions as (12), and Gamma priors are also placed on the hyperparameter γ to promote sparsity over the weights. However, it has been pointed out that, in contrast to RMV, Laplace priors placed on w can powerfully encourage sparsity while being log-concave [60]. In addition, the RVM can be considered a special case to be incorporated into the Laplace framework [58]. Therefore, the hierarchical Bayesian model using Laplace priors is employed in this study.

(12) $$p\left(w|\gamma \right)=\prod_{j=1}^{N}N\left(w_{j}|0,\gamma_{j}^{-1}\right)$$

Unlike RVM, the precision variables $\gamma_{j}^{-1}$ in (12) should be substituted by $\gamma_{j}$. To overcome the problem that the Laplace distribution is not conjugate to (10), $\gamma_{j}$ is assigned a hyperprior as [58]

(13) $$p\left(\gamma_{j}|\lambda \right)=\Gamma \left(\gamma_{j}|1,\lambda /2\right)=\frac{\lambda }{2}\exp\left(-\frac{\lambda }{2}\gamma_{j}\right),\gamma_{j}\geq 0,\lambda \geq 0$$

Then, the Laplace prior on w can be expressed as

(14) $$\begin{array}{ll} p\left(w|\lambda \right) & =\int p\left(w|\gamma \right)p\left(\gamma |\lambda \right)d\gamma =\prod_{j}\int N\left(w_{j}|0,\gamma_{j}\right)\Gamma \left(\gamma_{j}|1,\lambda /2\right)d\gamma_{j}\\ & ={\left(\frac{\sqrt{\lambda }}{2}\right)}^{N}\exp\left(-\sqrt{\lambda }\sum_{j}\left|w_{j}\right|\right) \end{array}$$

It has been demonstrated that the solution of (9) can be determined through the maximum a posteriori (MAP) estimation on w with (10) and (14), and λ plays the same role as the regularization factor κ [61,62]. Therefore, by adjusting the value of λ, the reconstruction algorithm under different sparsity constraints can be acquired, which is the theoretical basis of the adaptive hierarchical Bayesian model proposed in this study. Finally, the automatic estimation of λ can be achieved using the Gamma priors as follows:

(15) p(λ|τ)=Γ(λ|τ/2,τ/2)

**Figure 2:**
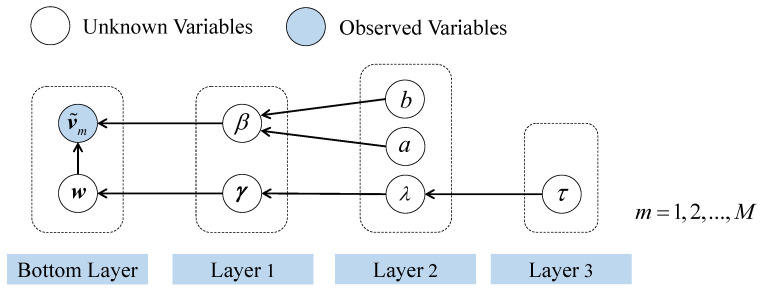
Graphical model of the Bayesian CS formulation using Laplace Priors.

To summarize, a four-layer hierarchical Bayesian model is built in this subsection, as shown in Figure 2. The observed vector $\tilde{v}$ and the sparse vector w constitute the bottom layer of the model. The introduction of the hyperparameters extends the model to higher stages, and the prior distributions act as links between layers. Combining the layers of the Bayesian model, the joint probability distribution can be obtained as follows (parameters a, b, and τ are omitted for clarity in this paper)

(16) $$p\left(\tilde{v},w,\gamma ,\lambda ,\beta \right)=p\left(\tilde{v}|w,\beta \right)p\left(\beta \right)p\left(w|\gamma \right)p\left(\gamma |\lambda \right)p\left(\lambda \right)$$

#### 2.3.2. DCS-Laplace with Parameter Estimation

**Figure 3:**
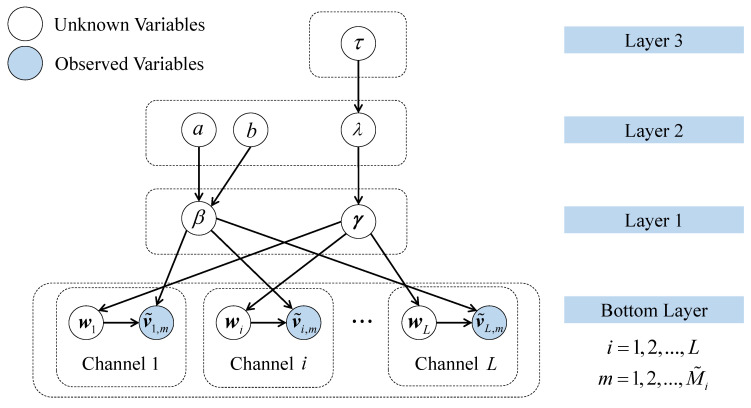
Hierarchical Bayesian representation of the DCS-Laplace.

To facilitate the joint reconstruction of multi-channel signals in (8), a multi-channel hierarchical Bayesian model using Laplace Priors, named DCS-Laplace, is extended from the original Bayesian CS formulation in this paper. As illustrated in Figure 3, the DCS-based approach enables the flexibility to model both the individuality of channels and information sharing among channels. Specifically, the channel-specific parameters ${\tilde{v}}_{i}$ and $w_{i}$ at the bottom layer are modeled through sparse learning with a unique ${\tilde{A}}_{i}$ in an individual channel, while the hyperparameters on the upper layers are fully shared to establish connections among channels. The common priors are determined by the joint learning of multi-channel signals, and the model in each channel learns independently while accepting the influence from other channels. As a result, the independent learning and joint learning of the reconstruction task are closely integrated to enable information transfer across channels.

Accordingly, in the proposed DCS framework, the prior distributions (10), (12), and (14) should be converted to

(17) $$p\left({\tilde{v}}_{i}|w_{i},\beta \right)=N\left({\tilde{v}}_{i}|{\tilde{A}}_{i}w_{i},\beta^{-1}\right)$$

(18) $$p\left(w_{i}|\gamma \right)=\prod_{j=1}^{N}N\left(w_{i,j}|0,\gamma_{j}^{-1}\right)$$

(19) $$\begin{array}{ll} p\left(w_{i}|\lambda \right) & =\int p\left(w_{i}|\gamma \right)p\left(\gamma |\lambda \right)d\gamma =\prod_{j}\int N\left(w_{i,j}|0,\gamma_{j}\right)\Gamma \left(\gamma_{j}|1,\lambda /2\right)d\gamma_{j}\\ & ={\left(\frac{\sqrt{\lambda }}{2}\right)}^{N}\exp\left(-\sqrt{\lambda }\sum_{j}\left|w_{i,j}\right|\right) \end{array}$$

To estimate the unknown parameters, the method of evidence maximization or type-II Maximum Likelihood is usually performed [59]. Firstly, try to maximize the posterior distribution as

(20) $$\prod_{i=1}^{L}p\left(w_{i},\gamma ,\lambda ,\beta |{\tilde{v}}_{i}\right)=\prod_{i=1}^{L}p\left(w_{i}|{\tilde{v}}_{i},\gamma ,\lambda ,\beta \right)p\left(\gamma ,\lambda ,\beta |{\tilde{v}}_{i}\right)$$

Since $p\left(w_{i}|{\tilde{v}}_{i},\gamma ,\lambda ,\beta \right)\propto p\left({\tilde{v}}_{i},w_{i},\gamma ,\lambda ,\beta \right)$, the distribution $p\left(w_{i}|{\tilde{v}}_{i},\gamma ,\lambda ,\beta \right)$ should also be a multivariate Gaussian distribution with mean and covariance

(21) $$\mu_{i}=\Sigma_{i}\beta {\tilde{A}}_{i}^{T}{\tilde{v}}_{i}$$

(22) $$\Sigma_{i}={\left[\Lambda +\beta {\tilde{A}}_{i}^{T}{\tilde{A}}_{i}\right]}^{-1}$$

where $\Lambda =\mathrm{diag}\left(\gamma_{j}^{-1}\right)$ considering that $p\left(\gamma ,\lambda ,\beta |{\tilde{v}}_{i}\right)\propto p\left({\tilde{v}}_{i},\gamma ,\lambda ,\beta \right)$, the hyperparameters can be estimated by maximizing the logarithm of the joint distribution $\prod_{i=1}^{L}p\left({\tilde{v}}_{i},\gamma ,\lambda ,\beta \right)$ with the marginalization over $w_{i}$, that is

(23) $$\begin{array}{ll} \left\{\gamma^{e},\lambda^{e},\beta^{e}\right\} & =\arg\underset{\gamma ,\lambda ,\beta }{\max}\sum_{i=1}^{L}\log p\left({\tilde{v}}_{i},\gamma ,\lambda ,\beta \right)\\ & =\arg\underset{\gamma ,\lambda ,\beta }{\max}\sum_{i=1}^{L}\log\int p\left({\tilde{v}}_{i},w_{i},\gamma ,\lambda ,\beta \right)dw_{i}\\ & =\arg\underset{\gamma ,\lambda ,\beta }{\max}\sum_{i=1}^{L}\log\int p\left({\tilde{v}}_{i}|w_{i},\beta \right)p\left(\beta \right)p\left(w_{i}|\gamma \right)p\left(\gamma |\lambda \right)p\left(\lambda \right)dw_{i} \end{array}$$

Referring to (21) and (22) and the solution of hyperparameters in (23), the unknown parameters can be estimated in an iterative process.

#### 2.3.3. An Efficient DCS-Laplace Algorithm with Modified Bayesian Model

Although the iterative algorithm discussed above is mathematically feasible, the computation complexity of $\mu_{i}$ and $\Sigma_{i}$ through (21) and (22) is extremely high [42,57,58]. This leads to sluggish calculation and numerical errors in the algorithm in the face of large-scale problems. Therefore, an efficient iterative approach is adopted in this study to improve its usability in practical engineering. In addition, to reduce the computational uncertainty caused by the initially estimated precision β, a modified sparse regression model for multi-channel signal reconstruction is introduced [42,45]. Instead of seeking the point estimates of β, the model integrates β out to concentrate on the iteration of hyperparameter γ.

In the modified Laplace framework, β is added to the prior distribution of the weights $w_{i}$ as (24), and all hyperpriors in (11), (13), and (15) are unchanged.

(24) $$p\left(w_{i}|\gamma ,\beta \right)=\prod_{j=1}^{N}N\left(w_{i,j}|0,\gamma_{j}\beta^{-1}\right)$$

Correspondingly, $p\left(w_{i}|{\tilde{v}}_{i},\gamma ,\lambda \right)$ is equivalent to $p\left(w_{i}|{\tilde{v}}_{i},\gamma ,\lambda ,\beta \right)$ integrating over β as

(25) $$\begin{array}{ll} p\left(w_{i}|{\tilde{v}}_{i},\gamma ,\lambda \right) & =\int p\left(w_{i}|{\tilde{v}}_{i},\gamma ,\lambda ,\beta \right)p\left(\beta |a,b\right)d\beta \\ & =\frac{\Gamma \left(a+N/2\right){\left[1+\frac{1}{2b}{\left(w_{i}-\mu_{i}\right)}^{T}\Sigma_{i}^{-1}\left(w_{i}-\mu_{i}\right)\right]}^{-\left(a+N/2\right)}}{\Gamma \left(a\right){\left(2\pi b\right)}^{N/2}{\left|\Sigma_{i}\right|}^{1/2}} \end{array}$$

With parameters

(26) $$\mu_{i}=\Sigma_{i}{\tilde{A}}_{i}^{T}{\tilde{v}}_{i}$$

(27) $$\Sigma_{i}={\left[\Lambda +{\tilde{A}}_{i}^{T}{\tilde{A}}_{i}\right]}^{-1}$$

To obtain the solution of hyperparameters, evidence maximization is utilized to perform Bayesian inference. The maximization of the logarithm of the joint distribution $\prod_{i=1}^{L}p\left({\tilde{v}}_{i},\gamma ,\lambda \right)$ can be expressed as

(28) $$\begin{array}{ll} \left\{\gamma^{e},\lambda^{e}\right\} & =\arg\underset{\gamma ,\lambda }{\max}ℒ\left(\gamma ,\lambda \right)\\ & =\arg\underset{\gamma ,\lambda }{\max}\sum_{i=1}^{L}\log p\left({\tilde{v}}_{i},\gamma ,\lambda \right)\\ & =\arg\underset{\gamma ,\lambda }{\max}\sum_{i=1}^{L}\log\int p\left({\tilde{v}}_{i}|w_{i},\beta \right)p\left(w_{i}|\gamma ,\beta \right)p\left(\gamma |\lambda \right)p\left(\lambda \right)p\left(\beta \right)dw_{i}d\beta \end{array}$$

The solutions of λ and τ from (28) are provided, respectively, as follows:

(29) $$\lambda =\frac{N-1+\tau /2}{\sum_{j=1}^{N}\gamma_{j}/2+\tau /2}$$

(30) $$\log\frac{\tau }{2}+1-\psi \left(\frac{\tau }{2}\right)+\log\lambda -\lambda =0$$

where ψ(τ/2) denotes the digamma function at τ/2.

The fast iterative algorithm adopted in this study originated from the work of Tipping et al. [63,64] and was extended afterward by Ji et al. [42], Babacan et al. [58], and Wang et al. [45]. The key to the approach is to convert the maximization of ℒ(γ) into the maximization of a separated component $l\left(\gamma_{j}\right)$ that only depends on a single variable $\gamma_{j}$. Therefore, the maximization can start with an empty vector (γ=0) and update a single $\gamma_{j}$ instead of updating the entire vector γ in each iteration, thus adding the components of the model one by one. Considering the sparsity of $w_{i}$, most of the $\mu_{i,j}$ and $\gamma_{j}$ will be set to zero, and the corresponding ${\tilde{A}}_{i,j}$ will be pruned out from the model. Hence, the matrix $\Sigma_{i}$ can be represented with fewer dimensions than N×N, leading to the greatly reduced computational complexity of the iterative algorithm. The update of the hyperparameters λ and τ at each iteration still follows the solution (29) and (30). The convergence criterion of the algorithm is defined as

(31) $$\frac{\left|\Delta ℒ\left(\gamma^{p}\right)-\Delta ℒ\left(\gamma^{p-1}\right)\right|}{\left|\max\left(\Delta ℒ\left(\gamma \right)-\Delta ℒ\left(\gamma^{p}\right)\right)\right|}<\varepsilon$$

where $\Delta ℒ\left(\gamma^{p}\right)$ is the increment of ℒ(γ) at the $p^{th}$ iteration, and ε is the predetermined threshold. For more details of the method, refer to Refs. [45,58]. The updated formulas of the parameters $\Sigma_{i}$ and $\mu_{i}$ are provided in Appendix B of Ref. [42].

## 3. Results

### 3.1. Case 1: Lieshihe Highway Bridge

**Figure 4:**
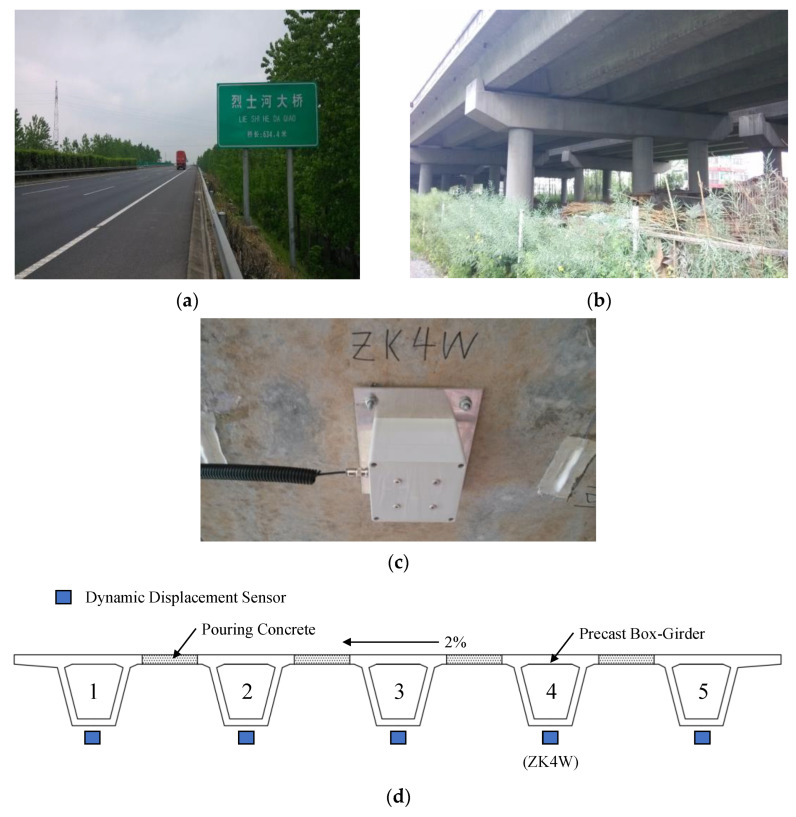
Lieshihe Highway Bridge: (**a**,**b**) the actual view; (**c**) site installation of sensor ZK4W; (**d**) sensor placement on the box girder.

Lieshihe highway bridge, shown in Figure 4a,b, is a representative continuous beam bridge on the Jiangsu Coastal Expressway in China. It is composed of two symmetrical lanes, left and right, with opposite traveling directions. The superstructure of each lane is composed of several 6×25 m prestressed concrete box girders bounded by expansion joints. An SHM system was installed to monitor the daily operation of the bridge in real time. As the typical vibration signal induced by traffic loads, the dynamic displacement data are used to simulate the wireless transmission in this study. The measuring positions are in the midspan of the girder with five sensors assigned to each lane. Taking the left lane as an example, the site installation of the sensor is shown in Figure 4c. Each sensor is deployed laterally at the bottom flange centerline of box girders 1–5, respectively, as presented in Figure 4d. The sampling frequency of the signal is 50 Hz.

**Figure 5:**
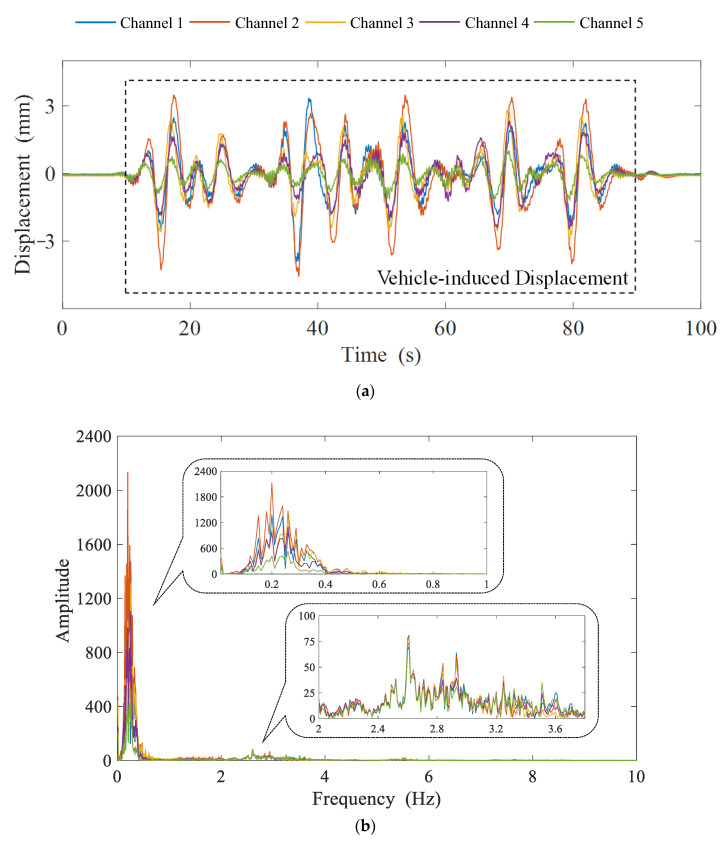
Dynamic displacement signals with 5 channels under vehicle events in the time and frequency domain, respectively: (**a**) the measured signals within 100 s; (**b**) the Fourier spectrum of the measured signals.

In this case, only signals from the left lane are used to simplify the research. Figure 5 shows an intercepted multi-channel signal at the same period in both the time and frequency domains. From Figure 5a, it can be observed that vibrations caused by passing vehicles will lead to the reciprocal displacement of the box girder, and the displacement will close to 0 when there are no vehicles. Figure 5b reveals that the Fourier amplitude of the signal from each channel, which is approximately sparsely distributed in the frequency domain, is mostly concentrated in the frequency interval of 0–0.5 Hz and 2–4 Hz with the negligible magnitude of the rest range. Meanwhile, the trend of the amplitude-frequency curves is quite close, indicating that the dynamic displacement data are strongly correlated in the frequency domain. The correlation matrix shown in Table 1 again confirms this fact. Therefore, the vehicle-induced dynamic displacement of the bridge girder is sufficiently sparse and intercorrelated, which meets the theoretical prerequisites of DCS. The vehicle-induced displacement in Figure 5a (10–90 s, 4000 data points in total) is finally selected as a case study of the DCS-Laplace algorithm for multi-channel signal transmission.

#### 3.1.1. Projection Matrix Setting and Data Loss Pattern

As mentioned earlier, vibration signals are compressed utilizing the RD-based projection matrix before transmission. Specifically, in the RD sampling process, the discrete-time signal will be randomly demodulated via the matrix D in the first step. Then the low-pass antialiasing filtering and downsampling are applied to the demodulated signal with the accumulate-and-dump operation of the matrix S [54], thus achieving data compression. Assume that a signal of 1 s is sampled at 12 Hz. Using RD to downsample at a low rate of 3 Hz, the corresponding sampling matrix can be expressed as

(32) $$S=\left(\begin{array}{llllllllllll} 1 & 1 & 1 & 1 & & & & & & & & \\ & & & & 1 & 1 & 1 & 1 & & & & \\ & & & & & & & & 1 & 1 & 1 & 1 \end{array}\right)=\left(\begin{array}{lll} b & & \\ & b & \\ & & b \end{array}\right)$$

where b is a sliding band with length l=4 and all elements equaling to 1. Define the compression ratio (CR) of the measurement vector as

(33) $$CR=\frac{N}{M_{i}}$$

It can be discovered that, after multiplying S in (29), the original signal will be encoded at CR=4. In the transmission process, the signal compression ratio and the redundancy of transmission loss can be regulated by adjusting the length and position of the sliding band in S [11]. For instance, suppose that the length of the sliding band is fixed at 4, to achieve the CR of 1, 2, and 4, respectively, the sampling matrix $S_{l,CR}$ can be expressed as follows:

(34) $$S_{4,1}={\left(\begin{array}{lllllll} 1 & 1 & 1 & 1 & & & \\ & 1 & 1 & 1 & 1 & & \\ & & \ddots & \ddots & \ddots & \ddots & \\ & & & 1 & 1 & 1 & 1\\ & & & & 1 & 1 & 1\\ & & & & & 1 & 1\\ & & & & & & 1 \end{array}\right)}_{N\times N}$$

(35) $$S_{4,2}={\left(\begin{array}{llllllllllll} 1 & 1 & 1 & 1 & & & & & & & & \\ & & 1 & 1 & 1 & 1 & & & & & & \\ & & & & 1 & 1 & 1 & 1 & & & & \\ & & & & & & \ddots & \ddots & \ddots & \ddots & & \\ & & & & & & & & 1 & 1 & 1 & 1\\ & & & & & & & & & & 1 & 1 \end{array}\right)}_{M_{i}\times N}$$

(36) $$S_{4,4}={\left(\begin{array}{lllllllllllllllll} 1 & 1 & 1 & 1 & & & & & & & & & & & & & \\ & & & & 1 & 1 & 1 & 1 & & & & & & & & & \\ & & & & & & & & 1 & 1 & 1 & 1 & & & & & \\ & & & & & & & & & & & & \ddots & & & & \\ & & & & & & & & & & & & & 1 & 1 & 1 & 1 \end{array}\right)}_{M_{i}\times N}$$

It can be observed that a proportion of redundant sliding bands appear in both $S_{4,1}$ and $S_{4,2}$, which can improve the robustness of missing data in the wireless transmission. However, the excessive pursuit of redundancy will inevitably lead to overlong sliding bands. This can not only increase the computational complexity but also the coherence of the projection matrix, which will lead to the degradation of the signal recovery [51]. Therefore, the length and position of the sliding band should be determined to balance the tradeoffs among the data compression, the tolerance of data loss, and the reconstruction performance. According to Refs. [11,51] and a series of trials in this study, the sliding band with l=4 was chosen for data compression. Note that when the original signal length N is not divisible by CR, it can be taken as $\hat{N}$, that is the smallest value of an integer multiple of CR greater than N, and then the column $N+1,N+2,\ldots ,\hat{N}$ of $S_{l,CR}$ should be deleted.

**Figure 6:**
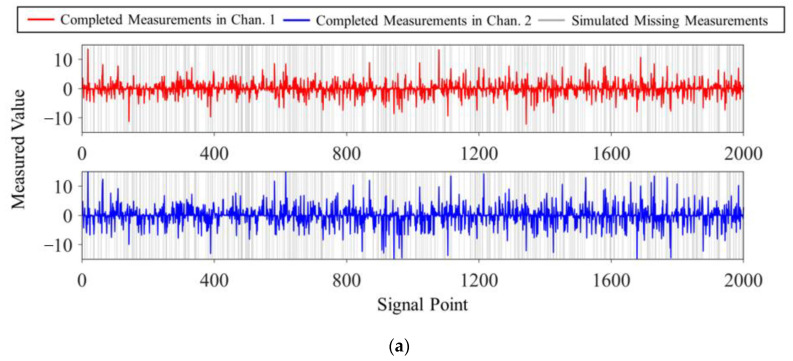

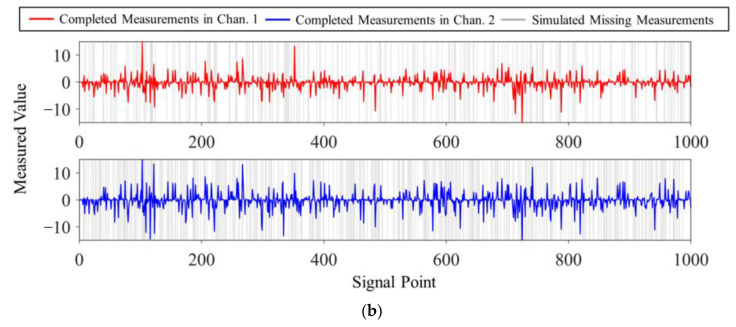
Received measurement vectors under different data loss patterns in Case 1: (**a**) uniform random loss, Channel 1 and 2 with CR=2, LR=20%; (**b**) non-uniform random loss, Channel 1 with CR=4, LR=20% and Channel 2 with CR=4, LR=40%.

The loss pattern of multi-channel signals can be classified as random loss and continuous loss. In the random loss pattern, each data packet of the measured vector $v_{i}$ would be lost randomly during wireless transmission. Continuous loss indicates that a stream of data packets of $v_{i}$ are lost during a certain time period of transmission. Moreover, in the proposed DCS framework, the data missing scenario (i.e., loss rate (LR) and loss sequence) varies from channel to channel. Since the permutation matrix **P** can equivalently convert the continuous loss pattern to the random loss pattern [51], the continuous data missing will not be considered in this study. To better present the performance of the reconstruction algorithm, signals with uniform random loss are used for algorithm testing. In addition, signals with non-uniform random loss are used to simulate the actual transmission situation in engineering and verify the applicability of the algorithm. The visualization of the data loss patterns in Case 1 is shown in Figure 6.

#### 3.1.2. Adaptive DCS-Laplace with Different Parameter Settings

In this section, the reconstruction performance of DCS-Laplace with different choices of the parameter λ is evaluated using the dynamic displacement data with uniform random loss, which focuses on both reconstruction accuracy and computation efficiency. To simulate the noise-free environment during wireless transmission, we set $\alpha =10^{-8}$ and b=1. The convergence threshold ε is taken as $10^{-8}$. To measure the reconstruction quality of the algorithm, the signal-to-noise ratio (SNR) is defined as

(37) $$SNR=20\log_{10}\left(\frac{{\left\|u_{i}\right\|}_{2}}{{\left\|u_{i}-{\hat{u}}_{i}\right\|}_{2}}\right)$$

where $u_{i}$ is the original signal and ${\hat{u}}_{i}$ is the recovered signal. Considering the randomness in data loss, each test is conducted 50 times and the results are averaged. All the experiments are performed on the same laptop platform programmed in MATLAB with an Inter (R) Core (TM) i7-10750 CPU @ 2.60 GHz. A series of DCS-Laplace algorithms with the following parameter settings are compared:

Algorithm 1-1 (Alg. 1-1): automatically estimated using Equation (26)
Algorithm 1-2 (Alg. 1-2): λ=0 (MT-BCS)
  Algorithm 1-3 (Alg. 1-3): λ=0.1
Algorithm 1-4 (Alg. 1-4): λ=1
Algorithm 1-5 (Alg. 1-5): λ=10

**Figure 7:**
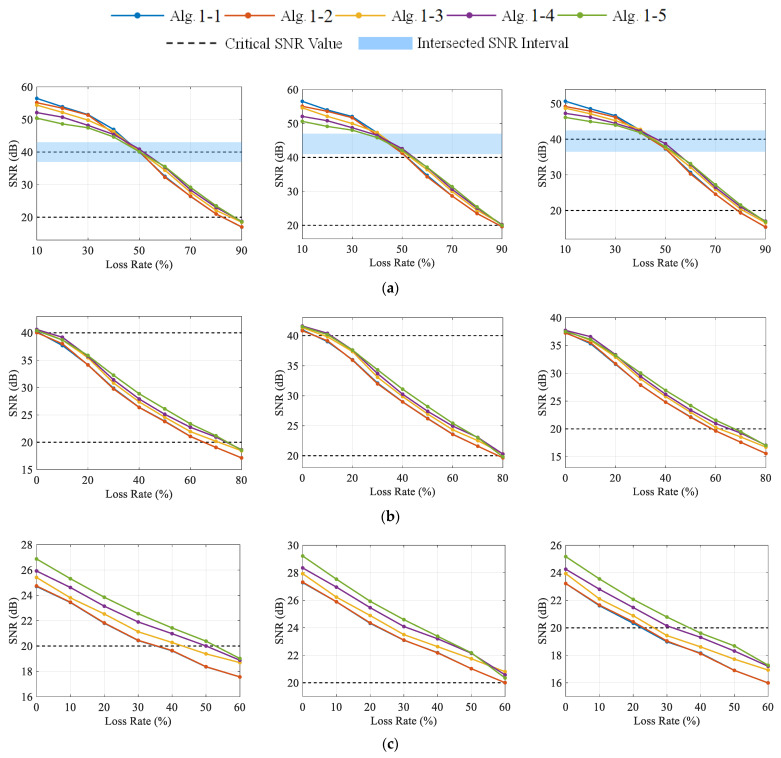
The SNR values of the recovered signal from channels 1–3 under different CR and LR with DCS-Laplace: (**a**) channels 1–3 from left to right with CR=1; (**b**) channels 1–3 from left to right with CR=2; (**c**) channels 1–3 from left to right with CR=4.

Figure 7 shows the reconstruction quality of the DCS-Laplace for vibration signals from channels 1–3 under different CR and LR, respectively. Taking channel 1 as an example, it can be observed that SNR-LR curves with CR=1 seem to intersect in a specific SNR interval. When the SNR value is higher than the interval, the reconstruction accuracy of Algorithm 1-2 to Algorithm 1-5 usually decreases with the increase in λ; when the SNR value is below the interval, the performance of Algorithm 1-2 to Algorithm 1-5 usually improves with the increase in λ. In fact, vibration signals in SHM are not strictly sparse in the frequency domain. As shown in Figure 4b, there will be a great number of non-zero entries close to zero in the sparse vector $w_{i}$. The increase in λ will strengthen the penalty on $w_{i}$, and more non-zero entries will be erased to zero, thus leading to the underfitting of the DCS-Laplace at low CR and LR (CR=1, LR≤40%). However, the rising sparsity of $w_{i}$ will improve the robustness of the data compression and transmission loss, especially at high CR and LR. Algorithm 1-1 automatically estimates the value of λ via (26) and outperforms all algorithms at low CR and LR, while its performance at high CR and LR is very close to that of algorithm 2. It should be noted that algorithm 2. with λ=0 is equivalent to the Multitask Bayesian Compressive Sensing (MT-BCS) with the framework of RVM proposed in Ref. [42].

**Figure 8:**
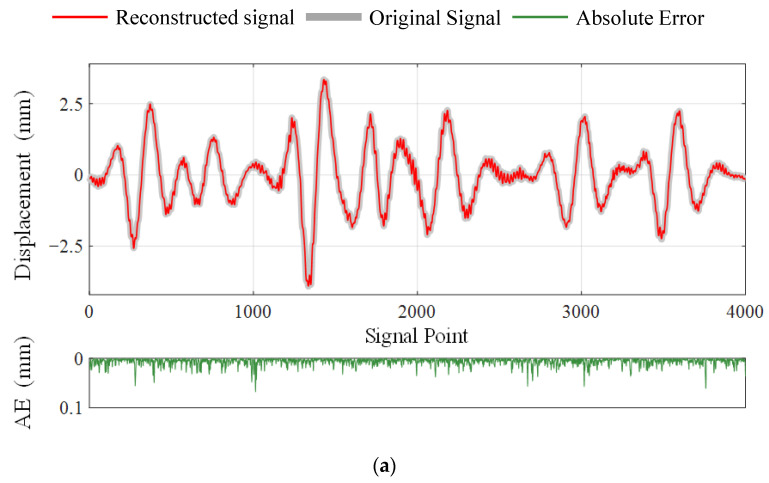

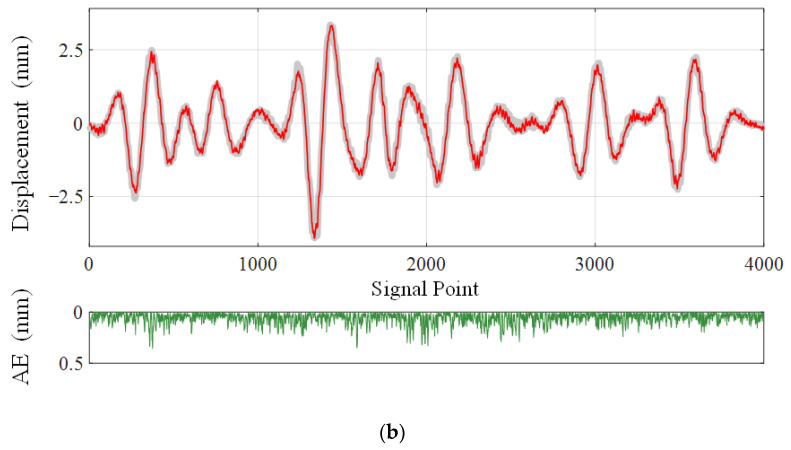
Reconstructed signals of channel 1 with AE when SNR values are close to 40 and 20, respectively: (**a**) SNR=39.8814; (**b**) SNR=20.3442.

Based on the above phenomena, the distortion of the recovered signal can be classified into three levels according to the SNR value, which are mild distortion (SNR>40), moderate distortion (40≥SNR≥20), and severe distortion (20>SNR). The critical values are marked with black dashed lines in Figure 7. Signals with mild distortion are very close to the original signals, which can meet the requirement of high-precision recovery. The moderately distorted signal has a high practical value as the accuracy can satisfy most engineering applications while providing enough redundancy for data compression and transmission loss. The SNR value of the reconstructed signal should be at least higher than 20; otherwise, it will be diagnosed as an unusable signal with severe distortion. Under this criterion, the proposed DSC-Laplace is an adaptive algorithm. By adjusting the penalty factor λ, DSC-Laplace can adapt to vibration signals with different sparsity to optimize performance. In Case 1, algorithm 1. is suggested if a high-accurate signal is required; algorithm 5. is suggested to obtain a signal with moderate distortion if striking the balance between transmission energy consumption, missing data allowance, and reconstruction quality. The reconstructed dynamic displacement of channel 1 with absolute errors (AE) when the SNRs are close to 40 and 20 is shown in Figure 8, respectively. According to the definition of SNR in (37), the decrease in the SNR indicates the accuracy decline of the reconstructed signal. Therefore, the AE of the reconstructed signal when the SNR is close to 20 is much higher than when the SNR is close to 40.

In addition, when comparing the SNR-LR curves from different channels at the same CR in Figure 7, it can be discovered that the curves of each algorithm follow the same trend, which again validates the performance variation among Algorithm 1-1 to Algorithm 1-5. Compared with channel 3, signals from channels 1 and 2 usually have higher SNR values under the same CR, LR, and algorithm choice. Therefore, signals from different channels can adjust the compression strategies according to their own characteristics to achieve high-quality wireless transmission.

In a test of computing efficiency, to simulate the reconstruction scenarios with different degrees of distortion, the CR of multi-channel signals are taken as 1, 2, and 4, while the LR is fixed at 30%. Table 2 shows the average computational time in a single reconstruction task. The results present that the recovery can be completed in a relatively short time (up to 127 s) for both mildly and moderately distorted signals, which meets the requirement of engineering applications. Moreover, the running time seems to be positively correlated with the reconstruction quality of the signal. As the CR increases, the SNR values and the running time under the same algorithm decrease significantly. At the same CR, the algorithm with higher accuracy usually requires a longer computation time, especially when CR=1 and CR=4.

#### 3.1.3. Performance Comparison of CS and DCS Methods

The following experiments are designed to verify the superiority of DSC over CS. According to the performance evaluation of the adaptive DCS-Laplace and the equivalent Bayesian method (MT-BCS), algorithms with automatically estimated λ and λ=10 are selected in this study. The original signals in CS algorithms are limited to single-channel signals, and the test environment is consistent with Section 3.1.2. Furthermore, the DCS-SOMP and its CS algorithm, which have recently been applied in SHM signal recovery [38,51], are also included to compare the performance with the Laplace method. Referring to the theorems of CS and RD [54,56], when applying the OMP method, stable recovery can be ensured if the sparsity of $w_{i}$ satisfies the following constraint at a certain CR and LR:

(38) M˜i≥C·Kilog(NKi)

where N is the length of the original signal, ${\tilde{M}}_{i}$ is the length of the received measured signal, $K_{i}$ is the number of nonzero components in $w_{i}$, and C is a positive constant depending on the tested signal. Based on the research in Refs. [11,54] and several trials in Case 1, the OMP method has sufficiently good performance when C=2. By eliminating the smaller coefficients in $w_{i}$, we chose the maximum nonzero number ${\hat{K}}_{i}$ that satisfies (35). Considering the similar trend of the SNR-LR curves for each channel, the reconstruction results except for channel 1 are omitted in this section. The tested algorithms are listed as follows:

Algorithm 2-1 (Alg. 2-1): DCS-Laplace with λ automatically estimated
Algorithm 2-2 (Alg. 2-2): DCS-Laplace with λ=10
Algorithm 2-3 (Alg. 2-3): DCS-SOMP
Algorithm 2-4 (Alg. 2-4): CS-Laplace with λ automatically estimated
Algorithm 2-5 (Alg. 2-5): CS-Laplace with λ=10
Algorithm 2-6 (Alg. 2-6): CS-OMP

**Figure 9:**
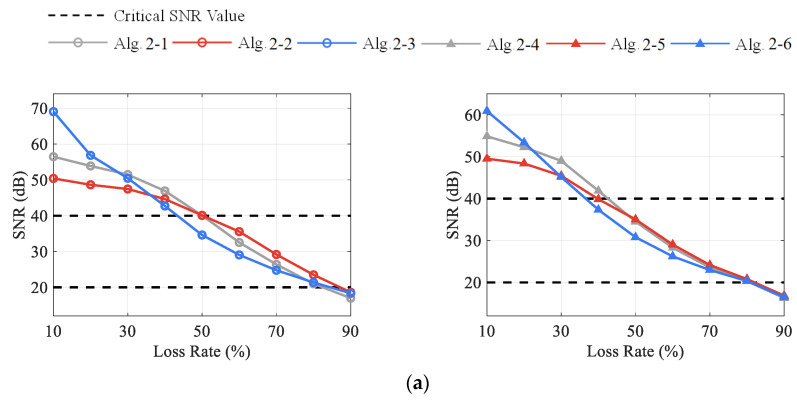

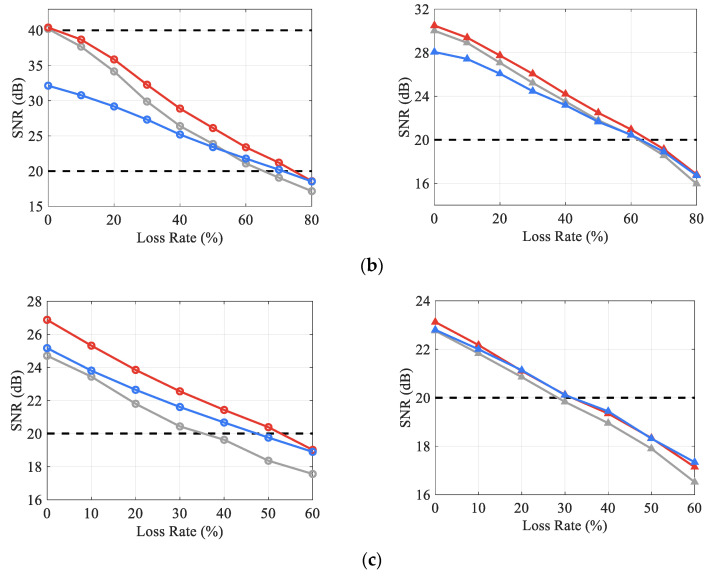
The SNR values of the recovered signal from channel 1 under different CR and LR with CS and DCS algorithms: (**a**) DCS vs. CS from left to right with CR=1; (**b**) DCS vs. CS from left to right with CR=2; (**c**) DCS vs. CS from left to right with CR=4.

Figure 9 presents the SNR-LR curves of the DCS Algorithm 2-1 to Algorithm 2-3 and the CS Algorithm 2-1 to Algorithm 2-6 using uniform random loss data. It can be discovered that, compared with the CS algorithms, DCS-Laplace and DCS-SOMP can increase the SNR values for both mild and moderate distortion signals under the same CR and LR, especially in the moderate distortion interval (40≥SNR≥20). This indicates that the DCS approach can effectively improve the reconstruction quality in the multi-channel signal transmission process. An interesting finding is that, with the same overall reduction, signals at higher CR usually have lower SNR, especially in the CS methods. For example, the SNR values of Algorithm 2-4 to Algorithm 2-6 with CR=1, LR=50% are higher than those when CR=2, LR=0%, although the signals in both cases are reduced by half. This is probably because the coding redundancy of the RD matrix goes down with the increase in CR, which leads to the recovery decline of CS [11,51]. However, the problem is alleviated in Algorithm 2-1 to Algorithm 2-3 as the frequency commonalities of the multi-channel signal are exploited in DCS. This phenomenon will not be further discussed as it is not the focus of this study.

Meanwhile, the performance difference between OMP and Laplace is also revealed in Figure 9. It can be found that the SNR values of OMP are higher than Laplace when only slight data loss occurs, regardless of CS or DCS methods. However, with the increase in CR and LR, the performance of Laplace gradually overtakes OMP, and the SNR gap reaches the maximum in the moderate distortion interval. Considering that signals are approximately lossless when SNR>40, the performance enhancement of OMP at low CR and LR is unnecessary in practical engineering. In contrast, Laplace can significantly raise the signal quality with moderate distortion. It is more advantageous when data compression is necessary or serious transmission loss occurs. Table 3 shows the calculation efficiency of Algorithm 2-1 to Algorithm 2-3 for a varying number of channels when CR=2, LR=0%, and Table 4 shows the average number of nonzero components in each recovery task. The computer configuration for the tests is the same as that in Section 3.1.2. As can be seen, the computing time gradually increases with the rise in channel number *L*. Compared with OMP, Laplace can complete the task in a shorter time even with more non-zero elements. Therefore, Laplace is a better choice in wireless signal transmission in terms of recovery performance.

#### 3.1.4. DCS Reconstruction in Non-Uniform Transmission Scenarios

To further demonstrate the applicability of DCS-Laplace to the proposed DCS framework in practical engineering, signals with non-uniform random loss are used to simulate the transmission situation. Different compression strategies are also adopted in multiple channels. Table 5 provides seven simulated scenarios. Taking Scenario 1 as a reference, the changing CR and LR in other scenarios are marked in orange. The corresponding results of DCS Algorithm 2-1 to Algorithm 2-3 are shown in Table 6. As can be seen, relative to Scenario 1, when the CR or LR of a single channel rises, the SNR values of itself, the adjacent channels, and even all the channels decrease; when the CR or LR of a single channel drops, the SNR values of itself, the adjacent channels, and even all the channels increase. This reveals that the reconstruction variation in a single channel can exert a positive influence on the remaining channels; Algorithm 2-1 to Algorithm 2-3 are proved to be effective in the proposed DCS framework, which can guarantee the independence of single-channel transmission while exploiting the inter-correlation among channels. However, it is important to note that the signal sparsity in OMP should be restricted by (35), which takes several influence factors, including CR, LR, and signal features, into account, comprehensively. This means that to obtain the optimal results for DCS-SOMP in Table 6, the recovery of each channel should be carried out separately, where the number of nonzero components ${\hat{K}}_{i}$ is set differently. This greatly limits the flexibility of the OMP method for multi-channel reconstruction tasks. In contrast, Laplace provides flexibility to enable both the individuality of channels and information sharing among channels, so it can achieve high joint-recovery quality despite the inconsistency of CR and LR across channels. In addition, by adjusting the penalty factor λ, Laplace can actively adapt to different reconstruction environments and reach peak performance conveniently. Therefore, DCS-Laplace also has higher applicability than DCS-SOMP in practical engineering.

In Table 5, the unit of LR is “%”; the orange background represents the changed CR or LR value referring to Scenario 1.

### 3.2. Case 2: Dashengguan High-Speed Railway Bridge

**Figure 10:**
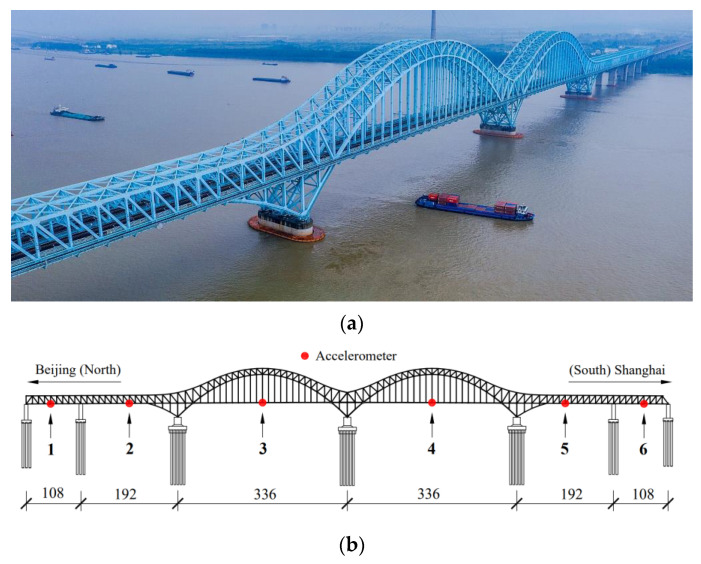
Dashengguan High-speed Railway Bridge: (**a**) the actual view; (**b**) accelerometer deployment on the bridge.

Dashengguan Yangtze River Bridge located in Nanjing is a six-line high-speed railway bridge. It was the largest high-speed railroad bridge in the world when it was completed, and is an essential part of the Beijing–Shanghai High-speed Railway. The superstructure of the bridge is a six-span continuous steel truss arch with a span arrangement of (108 + 192 + 336 + 336 + 192 + 108) m. The main bridge consists of three giant steel truss arches and steel bridge decks. A comprehensive SHM system has been installed on the bridge. To measure the vertical acceleration of the railway deck, six uniaxial accelerometers are employed separately in midspan sections of the main girder. The sampling frequency of the signal is 200 Hz. The bridge layout and the accelerometer placement are shown in Figure 10b.

**Figure 11:**
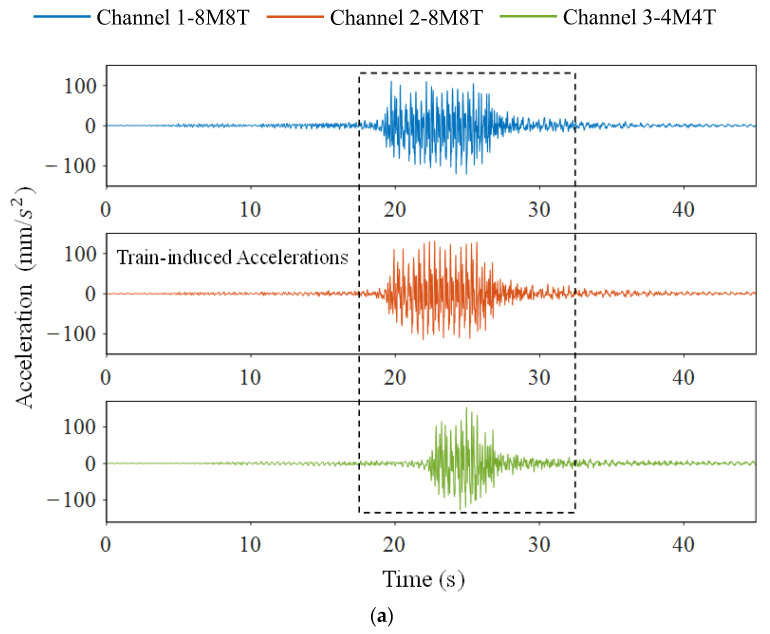

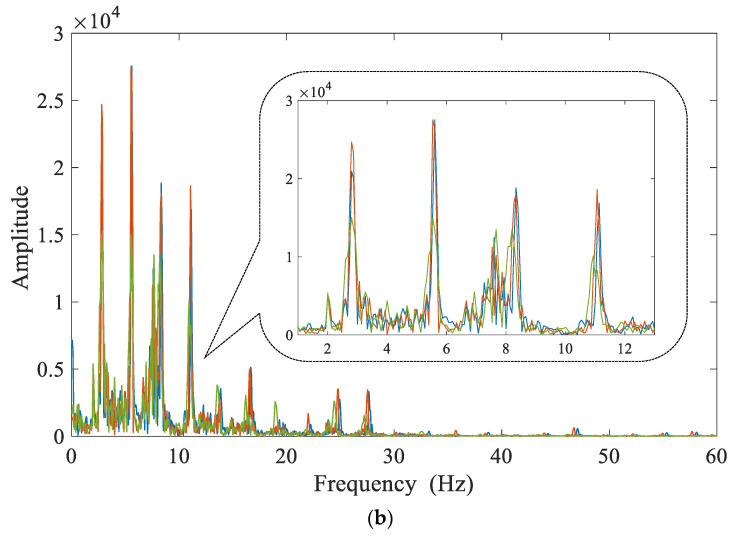
Acceleration signals from sensor 1 under 3 different train events in the time and frequency domain, respectively: (**a**) the measured signals within 45 s; (**b**) the Fourier spectrum of train-induced accelerations.

In China, the models of high-speed trains can be divided into 4M4T (four motors and four trailers) and 8M8T (eight motors and eight trailers) according to the train formation. The relatively uniform train models can lead to similar bridge–train interactions. Therefore, it can be speculated that accelerations at a fixed position of the girder under various train events may have a certain degree of correlation in the frequency domain. Figure 11a exhibits the vertical accelerations from sensor 1 when different trains pass over the bridge. To simplify the research, only three acceleration signals are selected, including two generated from 8M8T trains and one generated from the 4M4T train. The Fourier spectrums of train-induced accelerations (17.5–32.5 s, 3000 data points in total) are shown in Figure 11b, and the correlation matrix is also shown in Table 7. The results reveal that multi-channel accelerations under 4M4T and 8M8T train events are correlated in the frequency domain with sparse distribution, which meets the theoretical prerequisites of DCS. The RD matrix setting and data loss patterns remain the same, as in Case 1.

#### 3.2.1. Adaptive DCS-Laplace with Different Parameter Settings

In this section, the reconstruction performance of the adaptive DCS-Laplace is evaluated using acceleration signals with uniform random loss. The test environment is the same as Section 3.1.2. The DCS-Laplace with the following parameter settings are compared:

Algorithm 3-1 (Alg. 3-1): automatically estimated λ using (26)
Algorithm 3-2 (Alg. 3-2): λ=0 (MT-BSC)
Algorithm 3-3 (Alg. 3-3): λ=0.1
Algorithm 3-4 (Alg. 3-4): λ=1
Algorithm 3-5 (Alg. 3-5): λ=10

**Figure 12:**
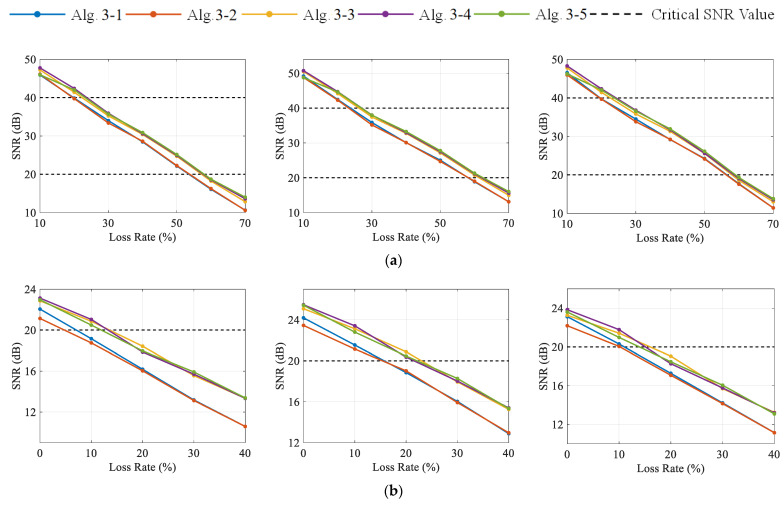
The SNR values of recovered signals from channels 1–3 under different CR and LR with DCS-Laplace: (**a**) channels 1–3 from left to right with CR=1; (**b**) channels 1–3 from left to right with CR=2.

**Figure 13:**
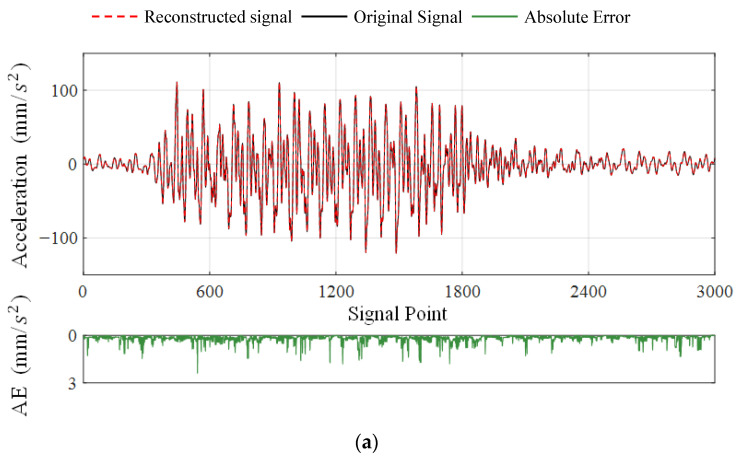

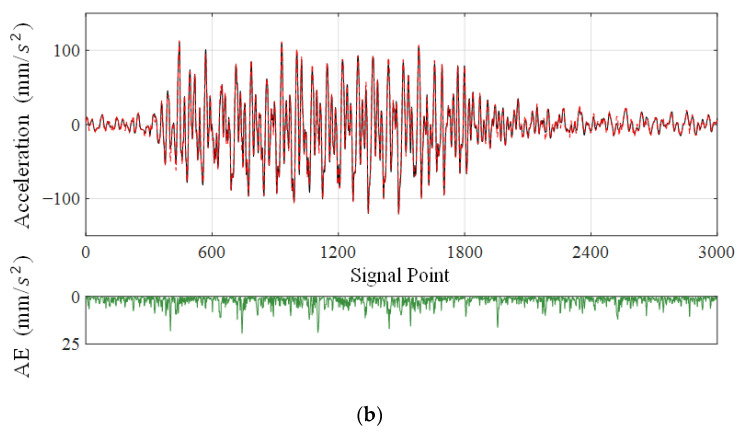
Reconstructed signals of channel 1 with AE when SNR values are close to 40 and 20, respectively: (**a**) SNR=40.5403; (**b**) SNR=20.0039.

Figure 12 illustrates the multi-channel SNR-LR curves of Algorithm 3-1 to Algorithm 3-5 with CR=1 and CR=2, respectively. In the instance of channel 1, it can be observed that the curves of algorithms 1-2 are very close to each other. The same phenomenon occurs in Algorithm 3-3 to Algorithm 3-5, and their SNR-LR curves are always above those of Algorithm 3-1 to Algorithm 3-2 (except when CR=1, LR=0%). This indicates that the reconstruction accuracy of Algorithm 3-3 to Algorithm 3-5 is higher than that of Algorithm 3-1 to Algorithm 3-2 in this case. In addition, the increase in λ does not lead to the obvious performance enhancement of Algorithm 3-4 to Algorithm 3-5. Instead, it causes the underfitting of mildly distorted signals at CR=1, LR=10% in Algorithm 3-5. Therefore, Algorithm 3-4 is suggested to acquire signals with mild and moderate distortions. Note that a higher CR is not desirable in this case as the acceleration under the high-speed train contains abundant high-frequency components, which results in its relatively low sparsity. In the face of high-frequency and low-sparsity vibration signals, the DCS in wireless signal transmission should concentrate on data loss recovery rather than a high degree of data compression. The reconstructed acceleration signals of channel 1 with an SNR close to 40 and 20 are shown in Figure 13, respectively.

Meanwhile, Table 8 shows the calculation efficiency of the Fast DCS-Laplace under different CR and LR. It can be discovered that the algorithm has a short computational time even for large-scale accelerations with low sparsity, and a single task can be completed basically within 300 s. Unlike Case 1, the positive correlation between the average running time and the reconstruction quality is not significant in this case, especially when CR=1, LR=10%.

#### 3.2.2. Performance Comparison of CS and DCS Methods

Additional simulations are carried out to verify the superiority of DCS over CS and compare the performance between the OMP and the Laplace method. In this case, the DCS-Laplace with λ=1 is chosen, and the constant C in (35) is also taken as 2 after trials. The rest of the experimental conditions remain the same as in Section 3.1.2. The tested algorithms are listed as follows:

Algorithm 4-1 (Alg. 4-1): DCS-Laplace with λ=1
Algorithm 4-2 (Alg. 4-2): DCS-SOMP
Algorithm 4-3 (Alg. 4-3): CS-Laplace with λ=1
Algorithm 4-4 (Alg. 4-4): CS-OMP

**Figure 14:**
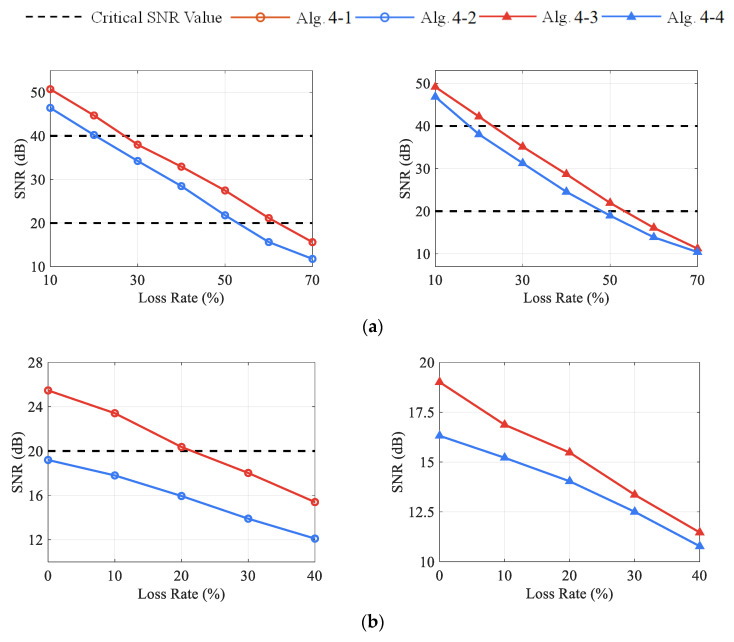
The SNR values of recovered signals from channel 2 under different CR and LR with CS and DCS algorithms: (**a**) DCS vs. CS from left to right with CR=1; (**b**) DCS vs. CS from left to right with CR=2.

Figure 14 shows the SNR-LR curves of DCS Algorithm 4-1 to Algorithm 4-2 and CS Algorithm 4-3 to Algorithm 4-4 at different CR and LR. Only the curves from channel 2 are provided to avoid duplicate outcomes. The results indicate that SNR values from DCS and Laplace are always higher than those from CS and OMP, respectively, at the same CR and LR. Meanwhile, the decrease in reconstruction accuracy due to the redundancy reduction in the RD matrix is reflected in both DCS and CS, especially in CS Algorithm 4-3 to Algorithm 4-4. Table 9 and Table 10 provide the average running time and nonzero element of the DCS Algorithm 4-1 to Algorithm 4-2 for different numbers of channels at CR=1, LR=25%. It can be found that the DCS-Laplace and DCS-SOMP have approximate computation efficiency: with a similar quantity of nonzero components, the running times of the two algorithms are close to each other. By comparing the results in Case 1, it can be speculated that different types of vibration signals can affect the computing efficiency of the DCS algorithms. Although beyond the scope of this study, future work could be conducted on this phenomenon. Table 11 and Table 12 exhibit the simulated non-uniform transmission scenarios and the comparison results of the DCS Algorithm 4-1 to Algorithm 4-2, which again demonstrates the strong applicability of DCS-Laplace in practical engineering.

In Table 11, the unit of LR is “%”; the orange background represents the changed CR or LR value referring to Scenario 1.

## 4. Conclusions

In this work, a comprehensive DCS framework for wireless signal transmission in SHM is constructed, incorporating the process of data compression and transmission loss together. Considering that common priors in the Bayesian framework can be set flexibly to facilitate multi-task information sharing, a hierarchical Bayesian model is developed for multi-channel signal reconstruction. To strengthen the sparsity constraint on vibration signals, Laplace priors are imposed on the sparse vectors. In addition, an efficient iterative algorithm based on a modified sparse regression model, called Fast DCS-Laplace, is adopted to guarantee its applicability in the face of large-scale problems. The reconstruction performance of the algorithm is tested using vibration data (e.g., dynamic displacement, accelerations) collected in real-world SHM systems and compared with the DCS-SOMP algorithm that has recently been applied in SHM. The main conclusions drawn are as follows:

- Facing multi-channel signals with similar sparse patterns, the DCS method can achieve joint recovery by exploiting the inter-correlation among channels, thus effectively improving the reconstruction performance. Even with a small number of channels (Case 2), DCS can still significantly improve the reconstruction quality and enhance the robustness of data compression and transmission loss compared with the single-channel CS approach. In addition, the proposed DCS framework also provides great flexibility and independence for single channels by using a unique sensing matrix in each task. The compression strategies of each channel can be adjusted according to its own characteristics to reach a compromise among the transmission energy consumption, the tolerance of data loss, and reconstruction accuracy, which is of high practical value in wireless signal transmission.
- DCS-Laplace is an adaptive algorithm that can actively adapt to different types of vibration signals by adjusting the constraints on sparsity to ensure the best reconstruction performance. In general, compared with the RMV-based hierarchical Bayesian model, imposing Laplace priors can achieve a higher reconstruction accuracy; the Fast DCS-Laplace algorithm can maintain a high operational efficiency in the face of large-scale vibration signals; the Laplace method has advantages over the OMP method in terms of reconstruction performance (especially for the reconstruction accuracy of moderately distorted signals) and applicability, which is a better choice in practical applications.

The proposed DCS framework can provide a flexible and practical multi-channel wireless transmission strategy for real-life SHM systems. The adopted DCS-Laplace algorithm plays an important role in the stages of data reconstruction, which can improve the robustness of SHM systems to data loss while facilitating data compression to reduce transmission demands.

## Figures and Tables

**Table 1:** Correlation Matrix of Fourier Amplitude Spectrum of Channels 1–5 in Figure 5b.

	Chan. 1	Chan. 2	Chan. 3	Chan. 4	Chan. 5
Chan. 1	1	0.9859	0.9374	0.9251	0.9039
Chan. 2		1	0.9555	0.9493	0.9214
Chan. 3			1	0.9685	0.9522
Chan. 4				1	0.9838
Chan. 5					1

**Table 2:** Computational time of Fast DCS-Laplace in Case 1.

	Alg 1-1	Alg 1-2	Alg 1-3	Alg 1-4	Alg 1-5
CR=1, LR=30%	126.9731 s	125.2586 s	114.4848 s	93.5504 s	83.0950 s
CR=2, LR=30%	54.0480 s	54.5392 s	49.5011 s	44.6238 s	48.6521 s
CR=4, LR=30%	10.1138 s	10.2370 s	11.8390 s	16.6280 s	22.2154 s

**Table 3:** Running Times of DCS Algorithms with varying *L* when CR=2, LR=0%.

	*L* = 1	*L* = 2	*L* = 3	*L* = 4	*L* = 5
Alg. 2-1	18.0238 s	39.5313 s	59.8162 s	81.0295 s	99.3569 s
Alg. 2-2	11.6737 s	26.6665 s	37.9230 s	60.1796 s	68.2221 s
Alg. 2-3	21.3301 s	46.6377 s	68.5796 s	91.5953 s	114.0725 s

**Table 4:** Average Nonzeros of DCS Algorithms for each task in Table 3.

	*L* = 1	*L* = 2	*L* = 3	*L* = 4	*L* = 5
Alg. 2-1	1441	1436	1497	1513	1482
Alg. 2-2	1073	1134	1126	1216	1172
Alg. 2-3	910	910	910	910	910

**Table 5:** Simulated Non-uniform Transmission Scenarios with Multi-channel Signals in Case 1.

	Scenario 1		Scenario 2		Scenario 3		Scenario 4		Scenario 5		Scenario 6		Scenario 7	
	CR	LR	CR	LR	CR	LR	CR	LR	CR	LR	CR	LR	CR	LR
Chan. 1	2	10	2	10	2	10	2	10	2	50	4	10	4	10
Chan. 2	2	30	2	30	2	30	2	30	2	30	2	30	2	30
Chan. 3	2	50	2	10	1	50	1	50	2	50	2	50	2	50
Chan. 4	2	0	2	0	2	0	2	0	2	0	2	0	4	0
Chan. 5	2	20	2	20	2	20	1	20	2	20	2	20	2	20

**Table 6:** Comparison Results of DCS Algorithms 2-1 to 2-3.

	Scenario 1	SNR (dB)				
		Chan. 1	Chan. 2	Chan. 3	Chan. 4	Chan. 5
DCS-Laplace (automatically estimated λ)	1	36.6662	34.0724	26.4272	32.6855	26.3189
	2	37.6487	35.4543	35.1345	33.2156	26.8240
	3	38.5772	35.1956	36.6870	34.1764	27.5615
	4	40.5286	34.6369	37.6548	35.5751	42.6754
	5	28.2025	32.7598	26.3141	31.3953	24.4993
	6	28.0049	32.6742	26.1043	31.0674	24.2534
	7	28.2131	31.9908	26.0608	27.0719	23.1887
DCS-Laplace (λ=10)	1	37.5008	36.3378	27.9808	33.6630	28.1540
	2	38.2697	37.1796	35.8453	34.0343	28.7652
	3	39.0819	37.6513	38.0546	35.0832	29.7045
	4	40.7152	37.9528	39.5789	36.1490	40.0842
	5	29.9361	35.1431	27.6651	32.5002	26.7678
	6	29.5179	34.7118	27.5555	32.3976	26.3908
	7	29.3773	34.0283	27.1823	28.1313	25.0307
DCS-SOMP	1	30.3471	31.1345	23.7890	28.1335	22.0260
	2	30.7220	31.3312	29.2418	28.5245	22.0624
	3	31.8760	32.0159	32.4429	30.0957	22.7680
	4	32.7611	31.9126	33.9947	32.3952	41.9091
	5	25.9234	30.4896	23.7146	27.6011	21.5107
	6	25.8473	30.5776	23.7111	27.6932	21.4629
	7	25.8201	30.2585	23.7947	25.1174	20.6950

**Table 7:** Correlation Matrix of Fourier Amplitude Spectrum of Channels 1–3 in Figure 11b.

	Chan. 1	Chan. 2	Chan. 3
Chan. 1	1	0.8790	0.7710
Chan. 2		1	0.8247
Chan. 3			1

**Table 8:** Computational time of Fast DCS-Laplace in Case 2.

	Alg. 3-1	Alg. 3-2	Alg. 3-3	Alg. 3-4	Alg. 3-5
CR=1, LR=10%	306.0228 s	293.1825 s	284.9227 s	249.9455 s	188.6743 s
CR=1, LR=40%	102.7295 s	100.3038 s	133.1828 s	129.5480 s	114.7767 s
CR=2, LR=10%	49.4826 s	47.9823 s	111.7089 s	135.2697 s	124.3077 s

**Table 9:** Running Times of DCS Algorithms with varying *L* when CR=1, LR=25%.

	Alg. 4-1	Alg. 4-2	Alg. 4-3
Alg. 1.	41.1258 s	107.1045 s	176.9771 s
Alg. 2.	44.1580 s	88.0031 s	133.1687 s

**Table 10:** Average Nonzeros of DCS Algorithms for each task in Table 9.

	*L* = 1	*L* = 2	*L* = 3
Alg. 1.	41.1258 s	107.1045 s	176.9771 s
Alg. 2.	44.1580 s	88.0031 s	133.1687 s

**Table 11:** Simulated Non-uniform Transmission Scenarios with Multi-channel Signals in Case 2.

	Scenario 1		Scenario 2		Scenario 3		Scenario 4	
	CR	LR	CR	LR	CR	LR	CR	LR
Chan. 1	1	10	1	10	1	50	2	10
Chan. 2	1	30	1	30	1	30	1	30
Chan. 3	1	50	1	10	1	50	1	50

**Table 12:** Comparison Results of DCS Algorithm 4-1 to Algorithm 4-2.

Algorithm	Scenario	SNR (dB)		
		Chan. 1	Chan. 2	Chan. 3
DCS-Laplace (λ=1)	1	47.3511	38.7360	26.1259
	2	47.2862	39.2743	47.3993
	3	25.1775	37.1179	25.9793
	4	21.4098	36.0167	24.6136
DCS-SOMP	1	43.4824	34.4658	20.2063
	2	43.6761	34.9629	43.7085
	3	19.1648	31.5436	20.0736
	4	16.5773	30.7059	19.3118

## Data Availability

All of the data, models, or codes that support the findings of this paper are available from the corresponding author upon reasonable request.
